# Polymer immobilized Cu(I) formation and azide-alkyne cycloaddition: A one pot reaction

**DOI:** 10.1038/srep09632

**Published:** 2015-05-12

**Authors:** Rafique Ul Islam, Abu Taher, Meenakshi Choudhary, Samarjeet Siwal, Kaushik Mallick

**Affiliations:** 1 Department of Chemistry, University of Johannesburg, Post Box 524, Auckland Park 2006, Johannesburg, South Africa

## Abstract

During the polymerization of aniline using copper sulphate, act as an oxidizing agent, the *in-situ* synthesized Cu(I) ion catalyzed the cyclo-addition between azides and alkynes. This work represents the merging of two steps, synthesis of the catalyst and application of the catalyst, in a one pot reaction. The elimination of the separate catalyst synthesis step is economic in terms of cost and time. As aniline was used as one of the reactant components so there is no requirement to use additional base for this reaction that further eliminates the cost of the process. Again, the catalyst can be readily recovered by filtration and efficiently used for the several sets of reactions without any significant loss of catalytic activity.

The cycloaddition between an azide and a terminal alkyne produce 1,2,3-triazoles are typical nitrogen-containing heterocyclic molecules that have attracted enormous interest due to their wide range of applications in biology^1^,^2^, medicinal chemistry^3^,^4^, design of new catalysts^5^ and also found wide industrial applications such as corrosion inhibitors, agrochemicals, optical brighteners, and photographic materials^6^. The cycloaddition process is based on a copper-catalyzed reaction protocol, which is highly regioselective to produce a 1, 4-disubstituted triazoles.

The azide-alkyne cycloaddition between an azide and a terminal or internal alkyne to give a 1,4- or 1,5-disubstituted 1,2,3-triazole, was developed by Rolf Huisgen^7^. The drawbacks of the Huisgen cycloaddition reaction are the requirement of high reaction temperatures and a lack of regioselectivity. Later, Sharpless^8^ and Meldal^9^ independently discovered that Cu(I) catalysts could facilitate the azide-alkyne cycloaddition in a regiospecific manner to give only 1,4-disubstituted triazoles.

Cycloaddition protocol was catalyzed with a Cu(I) source by using a Cu(I) salt^10^, CuSO_4_-ascorbate system^11^ and stabilized Cu(I) onto polymers^12^ or zeolite^13^. Copper nanoparticles^14^, metallic copper turnings^15^ and CuO nanoparticles^16^ have also successfully demonstrated activity for the title reaction. Cu_2_O is also a source of catalytic Cu(I) for azide-alkyne cycloaddition reactions. Applying Cu_2_O powder directly in a title reaction usually results an incomplete conversion and also require long reaction time^16^. Efforts have also been made to enhance the catalytic efficiency of Cu_2_O^17^,^18^,^19^,^20^. It is reported that^17^ polyvinylpyrrolidone-coated Cu_2_O nanoparticles can act as an efficient catalyst for cycloaddition reactions in water at physiological temperature. The results in this paper indicated that Cu_2_O-NPs were less toxic than the commonly used CuSO_4_-reductant catalyst systems^21^,^22^. Polymers have the potential benefit as a support of the catalyst for a wide range of applications^23^,^24^,^25^,^26^ due to the combination of both robust and flexible nature^27^.

Scientists has given attention to develop the catalysts for the synthesis of 1,2,3-triazoles in such a way so that Cu(I) efficiently catalyzed the reaction under mild conditions to give 1,4-disubstituted 1,2,3-triazoles. In connection with our on-going research on the development of effective catalysts for synthetic organic transformations^27^,^28^,^29^,^30^,^31^,^32^, we have found that a polyaniline supported Cu(I) supramolecular composite system can be used for the azide-alkyne cycloaddition reaction where heterogeneous catalyst could be easily separated from the crude reaction mixture and recycled in a given process.

In recent years, the environmental aspects such as atom efficiency, waste production and energy consumption are very important issues for consideration of a chemical reaction. In this regard, the combination of two or more synthetic steps into one operation is a very appealing methodology since time, energy and resources consuming workup and purification steps can be minimized. Considering the above facts, in this present communication we report a convenient one pot method for the synthesis of polymer stabilized Cu(I) catalyst and Cu(I) catalyzed azide-alkyne cycloaddition reaction under ambient condition. In the reaction pot, polymer stabilized Cu(I) catalyst was formed due to the ‘*in-situ* polymerization and composite formation’ (IPCF) reaction^33^,^34^,^35^,^36^,^37^,^38^.[Fig f1]

## Result and discussion

### Polymer immobilized Cu(I) formation (Figure 1): Proof of evidence

**Figure 1 f1:**
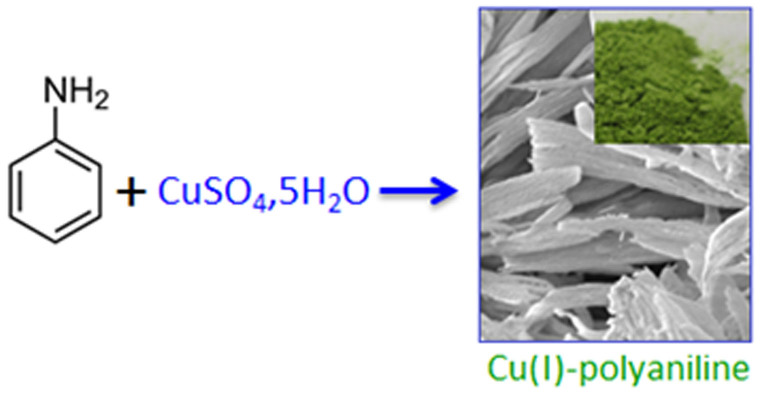
Polyaniline immobilized Cu(I) formation.

**Figure 2:**
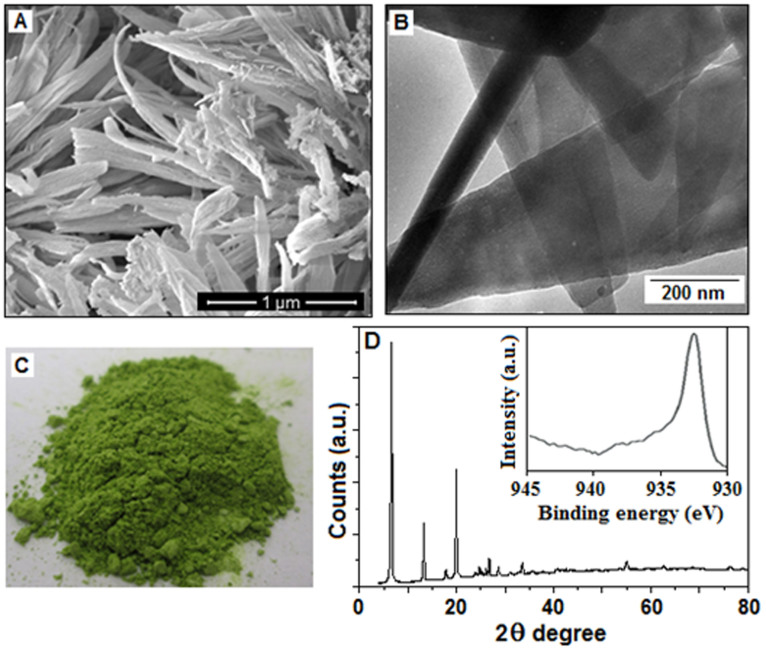
The SEM image (A) of the Cu(I)-polyaniline complex whereas the TEM image (B) of the polymer and no evidence of the formation of copper nanoparticles has been observed in the image. (C) The Cu(I)-polyaniline composite material (dried). (D) The XRD pattern indicates the crystalline character of the polyaniline, there being no indication for the formation of the metallic copper. X**-**ray photoelectron spectroscopy (XPS) analysis shows (in-site) the high intensity peak at 932.5 eV could be assigned to the binding energies of *Cu* 2*p*_3/2_, indicating the presence of Cu (I).

In a typical experiment, aniline monomer (5.0 mM) was diluted in methanol in a conical flask and an aqueous solution of CuSO_4_, 5H_2_O (10^−2^ M) was added drop-wise (1:2 molar ratio of copper sulphate to aniline) to it under stirring condition. During the addition, the solution took on a green colourization and at the end a parrot green precipitation was formed at the bottom of the conical flask. The entire reaction was performed at room temperature and under open atmosphere. Here, IPCF synthesis technique has been followed for the preparation of a Cu(I)-polyaniline supramolecular composite material using copper (II) sulphate as an oxidizing agent for polymerizing aniline. During the polymerization process each step is associated with a release of electron and that electron reduces the Cu^2+^ ion to form Cu^+^ ion. The Cu^+^ ion binds with the chain nitrogen of the polyaniline to form an N→Cu(I) type of bond, where polymer acts as a micro ligand. The SEM image (Figure 2A) illustrates the fiber-like morphology of the Cu(I)-polyaniline complex. The TEM image (Figure 2B) shows the surface morphology and internal microstructure of the polymer. A thin area of the sample was selected for viewing and acquiring the TEM images. It is clear from the TEM image that the surface is very smooth as well as transparent and has no evidence for the presence of copper nanoparticles. Figure 2C represents the colour of the resultant dried sample. The sample was also characterized with X-ray diffraction (XRD) analysis (Figure 2D). The XRD pattern confirms the crystalline character of the polyaniline and there is no indication for the formation of the metallic copper. To confirm the valence state of copper present in the sample X-ray photoelectron spectroscopy (XPS) analysis was done. A high intensity peak at 932.5 eV could be assigned to the binding energies of Cu (I) (Figure 1D, in-set). No characteristic peaks are identified for Cu (II) and Cu (0), suggesting that copper (II) precursor is converted to Cu (I).

**Figure 3:**
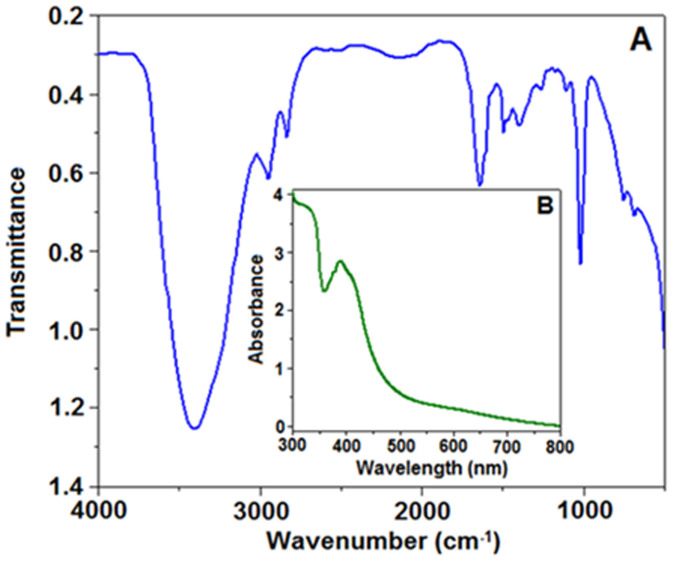
Fourier transform infrared (FT-IR) spectrum (A) of the resultant material showing the presence of benzenoid and quinoid rings at 1496 and 1638 in the polymer, respectively. The UV-vis spectrum (B) of Cu-polyaniline show a shoulder-like appearance at about 330 nm corresponds to π-π* transition of benzenoid rings and at about 400 nm a prominent peak represents polaron/bipolaron transition.

Figure 3 shows the optical characterization of the resultant Cu(I)-polyaniline composite. The IR analysis of the fingerprint region is useful for examining the resonance modes of the benzenoid and quinoid units of polyaniline. In the IR spectra (Figure 3A), the peak at 1638 cm^−1^ corresponds to the group N = Q = N (where Q represents a quinoid ring), while the N-B-N group (where B represents a benzenoid ring) absorbs at 1496 cm^−1^. The N-H stretching mode at 3400 cm^−1^ has been identified for the Cu(I)-polyaniline sample. These results are in good agreement with previously reported spectroscopic characterizations data of the polyaniline^39^. The intensity of the peak for quinoid ring structure is higher indicates that the polymers are higher in oxidation state. The UV-vis spectrum (Figure 3B) of Cu-polyaniline show a shoulder-like appearance at about 330 nm corresponds to π-π* transition of benzenoid rings (inter-band transition) and at about 400 nm a prominent broad peak represents polaron/bipolaron transition. A weak absorption band with a curvilinear behaviour has been observed within the range of 500–700 nm indicates the benzenoid to quinoid excitonic transition in both the polymers^40^. All the above microscopic and spectroscopic characterization techniques proved the formation of Cu(I)-polyaniline during the reaction between aniline and copper sulphate.

### Polyaniline supported Cu(I) formation and azide-alkyne cycloaddition

**Figure 4:**
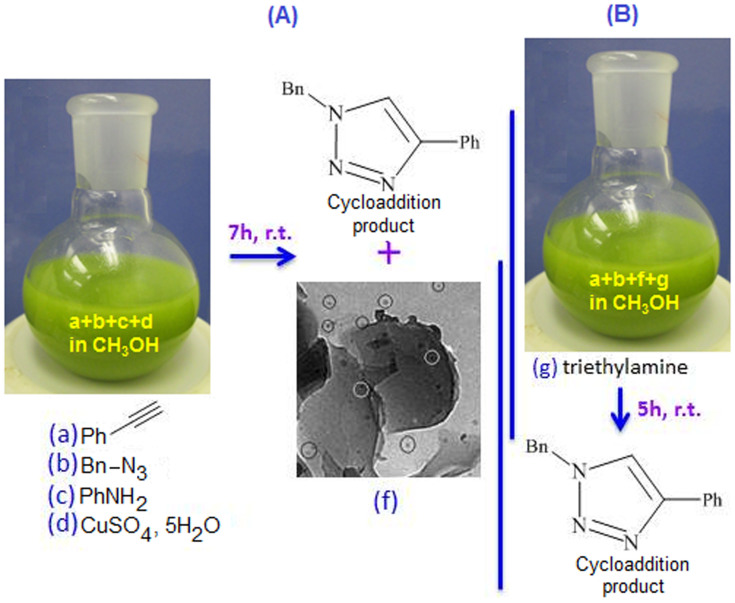
(A) Cycloaddition reaction between azide and alkyne in presence of aniline and copper sulphate using methanol as a solvent. (B) The recyclability study of the azide and alkyne cycloaddition reaction using Cu-polyaniline composite recovered from the reaction mentioned in Method 1, Figure 4 (A). All the reactions were done under room temperature (r.t.).

After confirmation of the formation of the Cu(I) species we have followed the procedure mentioned in ‘Method: 1’ for the cycloaddition reaction between azide and alkyne (Figure 4A).

The 1,3-dipolar cycloaddition reaction has been tested using benzyl azide, **1a**, with phenyl acetylene, **2a**, for the synthesis of di-substituted 1,2,3-triazoles, **3a**, at room temperature under different solvent conditions such as dichloromethane, chloroform, toluene, ethanol, methanol, water and methanol : water (1:1) mixture in the presence of copper sulphate and aniline. Among the above solvents, methanol and the combination of methanol-water system gave the highest product conversion, product yield 99% for the period of 7h (Table 1). Considering the above results we have decided to use methanol as a solvent for the rest of the study to ease the work-up procedure. Due to the basic nature of aniline, in this study we did not add any external base as per recommendation for the 1,3-dipolar cycloaddition reaction^41^. The best result was achieved when the catalyst concentration was 3.0 mol% Cu (on the basis of the amount of aniline present in the reaction mixture and also considering all the aniline to be converted to polyaniline as a support). By increasing the amount for Cu concentration, no further improvement of the reaction has been identified in terms of time (Table 1, entry 6). Besides that, the reaction between benzyl azide with acetylene was also carried out in the presence of Et_3_N under the same reaction condition to find out the significance of Et_3_N in the reaction. We have observed the presence of Et_3_N delayed the reaction significantly may be due to the coordination between Et_3_N and copper sulphate forms relatively stable intermediate complex, [Cu(NEt_3_)_4_]^2+^, which require more energy to breakup and for the participation of the reaction^42^. The product, 1-benzyl-4-phenyl-1*H*-1,2,3-triazole **(3a)**, was characterised by spectroscopic method and found to be identical with the previously reported one^43^.

Based on the above optimized reaction condition, we have explored the versatility of the *in-situ* generated catalyst for the 1,3-dipolar cycloaddition of various azides and alkynes and the results are summarized in Tables 2. In this study, we also have used structurally diverse azides and alkynes. All the substrates produced the expected cycloaddition product with very good to excellent yields and selectivity. Phenylacetylene and its derivatives (Table 2, entries 1–3) gave a higher isolated yield when coupled with azides. It was found that the yield was as high as 99% for the coupling of benzyl azide with phenylacetylene (Table 2, entry 1). When benzyl azide coupled with phenylacetylene with electron withdrawing and donating groups no such noticeable difference has been observed in terms of yield for the cycloaddition product (Table 2, entries 2 and 3 respectively). Alkyne attached with heteroaromatic molecule afforded the product 1-benzyl-4-(thiophen-3-yl)-1*H*-1,2,3-triazole when coupled with benzyl azide and a decrease of yield has been observed in comparison with the aromatic substituted molecules (Table 2, entry 4). Cycloaddition between aliphatic alkynes and benzyl azide (Table 2, entries 5–8) is comparatively less efficient than alkynes attached with aromatic and heteroaromatic molecules. The cycloaddition of 2-bromobenzyl azides (bromine substituted benzyl azide) with different alkynes (Table 2, entries 9–16) shows an identical reactivity trend that found for the benzyl azide (Table 2, entries 1–8). All the above products have been achieved over the period of 7 h under the ambient atmospheric condition.

### Performance of the recovered catalyst

In heterogeneous catalysis, the durability of the catalyst is an important issue from the economic and sustainability point of view.

**Figure 5:**
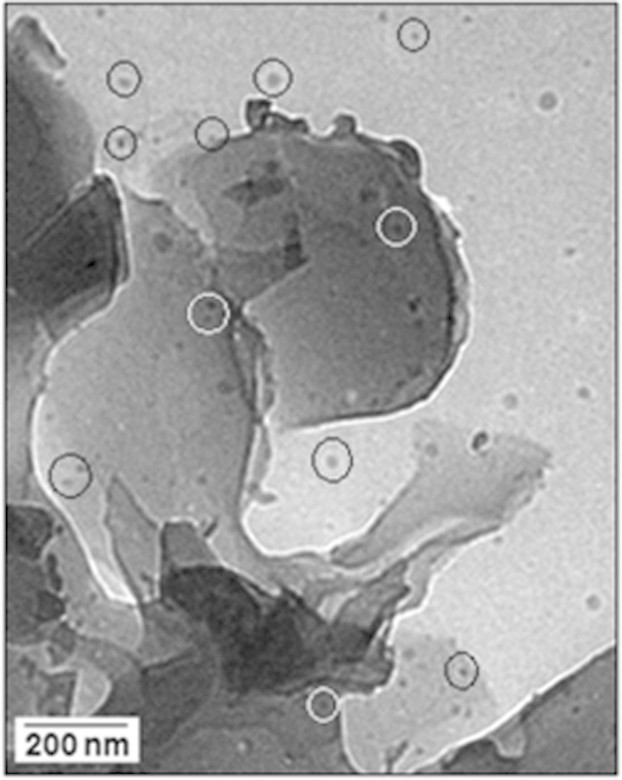
The TEM image of the used catalyst (after the end of the first cycle) showed the formation of copper nanoparticles (some of them are indicated within circles) with a wide range of size distribution.

To study the performance of the recovered catalyst, for the reaction mentioned in Table 2, entry 1, we have increased the amount of the reactants by a factor of 10 (for convenience, the concentration of the copper sulphate has been changed to 0.1 mol dm^−3^) and monitored the reaction using thin layer chromatography technique. After completion of the reaction, which took about 7 h, the product (**3a**, as confirmed by spectroscopic analysis and with a yield of ~98%) was extracted and the other product, Cu-polyaniline, was separated. The stability and recyclability performance of the *in-situ* synthesized, Cu-polyaniline, was tested as a catalyst for the above cycloaddition reaction using the following procedure, Figure 4B. Alkyne (**1a**) and azide (**2a**) were mixed in the presence of methanol and to this solution triethylamine and recovered Cu-polyaniline catalyst were added. In the cycloaddition reaction, the role of triethylamine is to activate the acetylenic proton to form the phenyl acetylide which further react with the copper catalyst to form copper acetylide. Copper acetylide then reacted with azide to form trizole derivative. Whereas, in one pot reaction aniline performed the role of base and no need to use an external base like triethylamine. The recovered catalyst (Cu-polyaniline) was also characterized by TEM. The presence of the copper nanoparticles was clearly noted with a wide range of size distribution (10–40 nm) on the polymer matrix (Figure 5). So far as the nanoparticles are concerned, the surface of the particles is considered to be more reactive as a catalyst and the present study revealed the similar experience during the reaction process. A yield of 98% of the coupled product (**3a**) has been achieved for the reaction between **1a** and **2a** and that took about 5 h, which is two hours less than the original single pot reaction, indicates the catalytic effect of the nanoparticles. At the end of the fifth cycle, a yield of 87% of cycloaddition product was achieved at about 5 h. The recyclability study has also been performed using the recovered catalyst in the absence of base (NEt_3_) and only 53% of the product has been achieved under the same reaction condition for 7h.

**Figure 6:**
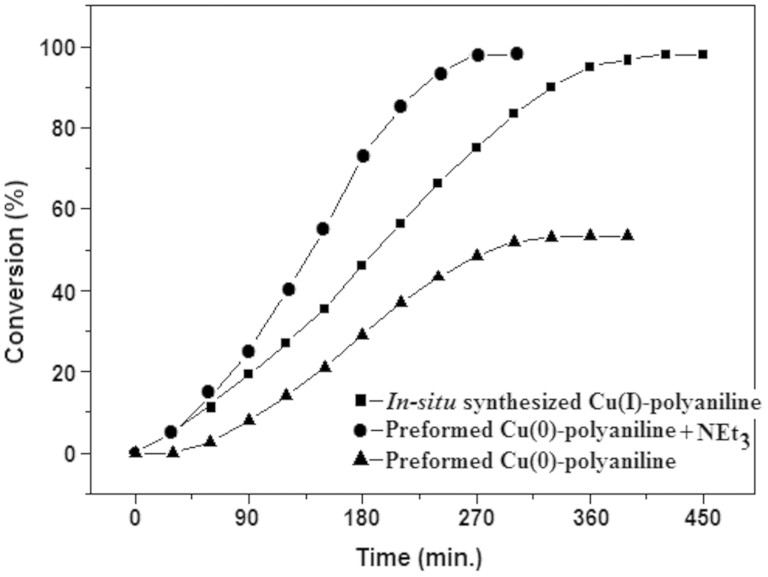
Comparative kinetic study of the cycloaddition reaction between benzyl azide and phenyl acetylene using *in-situ* synthesized Cu(I)-polyaniline catalyst (▪) and preformed Cu(0)-polyaniline catalyst in the presence (●) and in the absence (▴) of triethylamine.

We have also performed the kinetic studies of the cycloaddition reaction (Table 2, entry 1) for the (1) *in-situ* reaction, (2) reaction where the recovered Cu-polyaniline was used as a catalyst in presence of base and also (3) for the reaction using recovered Cu-polyaniline as a catalyst in absence of the base. The results are shown in the graph (Figure 6). From the graph it is clear that the recovered catalyst is more active in presence of a base than the *in-situ* synthesized catalyst but for the first 30 min of the reaction an identical amount of product (~5% of the yield) has been achieved for the first two reactions. So, from the kinetic study it is confirmed that Cu(I) and Cu(0) are the catalyst species, for the cycloaddition reaction between organic azides and terminal alkynes, for the reaction (1) and (2), respectively, and it is also evident from the recyclability study that the catalytic activity of copper nanoparticles are higher than copper (I). The results are also supported by the previously reported literature^44^. For the reaction using preformed Cu(0)-polymer as a catalyst in absence of base (3), the reaction was slow, only ~5% product has been formed in the first 60 min of the reaction and total 53% product has been achieved at the end of the reaction.

Various sources of the active Cu(I) catalyst for the alkyne-azide cycloaddition has been reported. Cu(II) sulphate has also been successfully used as a catalytic precursor in the presence of sodium ascorbate to generate the catalytically active Cu(I) species^45^. The Cu-carbon catalyst using charcoal and Cu(NO_3_)_2_ as the precursor in presence of water as a solvent works very efficiently for the title reaction^46^. Both Cu(I) and Cu(II) oxide show the catalytic activity for the synthesis of 1,2,3-triazole products in the multicomponent click synthesis under ambient conditions^43^. There is also an evidence of direct participation of Cu(II) for the synthesis of 1,2,3-triazoles using high catalyst loading in aqueous solutions for 20 h^47^, indicates Cu(II) may not be an efficient solution for alkyne-azide cycloaddition reaction. We found that the use of only CuSO_4_, 5H_2_O as a catalyst need more than 24 h to achieve a 55% yield of the cycloaddition product between azide and alkyl in presence of excess base.

For the synthesis of the desired compound, metal contamination in the product is a matter of serious concern^48^. Leaching of the catalyst into the product would implicate a time-consuming and costly process, which would make the whole process more expensive. Several methods have been developed to distinguish between soluble and insoluble catalysts^49^ and some of these methods were also used for the current study in order to investigate whether the solid catalyst is heterogeneous or not.

As our study was carried out at ambient temperature so room temperature filtration test was performed. During this test, the catalytically active species were removed from the reaction mixture by filtration and the filtrate was monitored for catalytic activity. It was observed that after removal of the catalyst; the reaction did not proceed, indicating that no catalytically active copper remained in the filtrate. However, the filtration test alone cannot prove the heterogeneous nature of the reaction as the leached metal species may not be sufficient enough to show the catalytic performance. To confirm that, the reaction supernatant was analysed by ICP-MS (Inductively coupled plasma mass spectrometry) technique, a type of mass spectrometry which is capable of detecting metals at concentrations as low as one part in 10^12^ (part per trillion) level, and no detectable amount of copper species was found in the solution suggest a heterogeneous mechanism for the cycloaddition reaction using Cu(I)-polyaniline as a catalyst.

### A single pot multicomponent reaction both for Cu(I) catalyst formation and azide-alkyne cycloaddition

**Figure 7:**
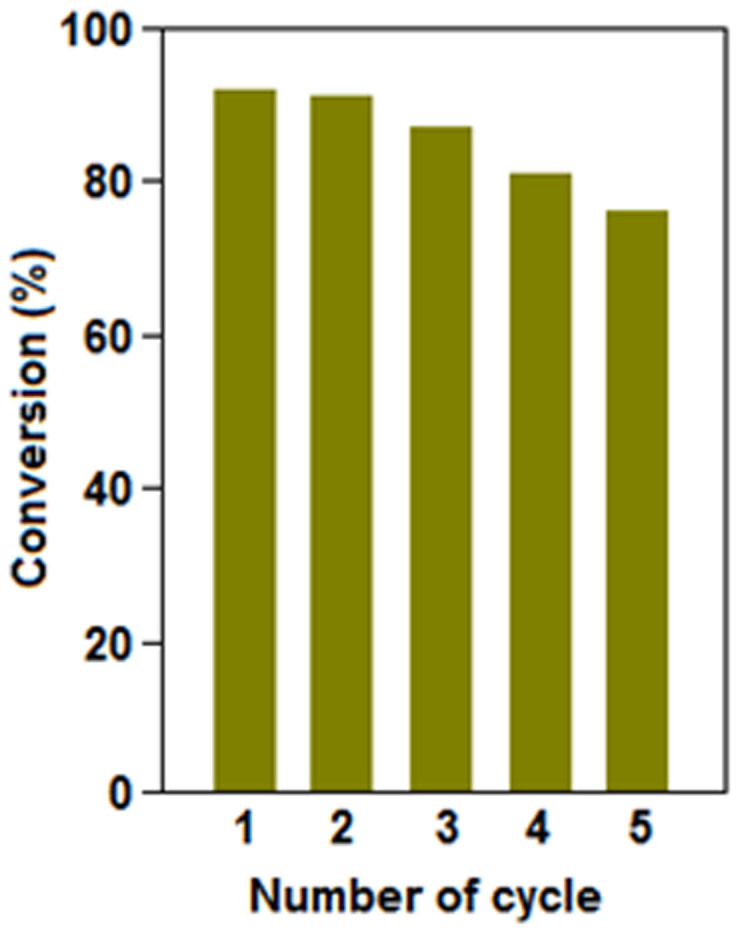
Recyclability study of the catalyst (preformed Cu(0)-polyaniline in the presence of triethylamine) was tested for the reaction mentioned in Table 3, entry 1.

Most of the copper catalysed azide-alkyne cycloaddition reports are on two component (organic azide and alkyne) reaction systems. In the two component synthesis method, the organic azides need to be synthesized in advance and the isolation process can be problematic. It is thus desirable to develop an efficient one-pot methodology that uses alkyl halides and sodium azide for direct cycloaddition with alkynes in the presence of suitable catalyst. Multicomponent reactions have many advantages in comparison with multi-step reactions according to environmental and economic considerations. Therefore, the design of novel multicomponent system has attracted a lot of attention from research groups working in various areas of organic synthesis. In the present work, we also turned our attention towards the one-pot, three-component Click reaction (Table 3) in which the azide-alkyne cycloaddition products were generated *in-situ* from their precursor, aryl bromides, sodium azide and alkyne, by minimising one step. The presence of aniline and copper sulphate in the multicomponent system acts as the precursor of Cu(I)-polyaniline catalyst in presence of methanol as a solvent for the period of 9 h to give the desired products (Table 3, entries 1–6) with the isolated yields ranging from 81–92% (Method 2). To perform the recyclability test of the catalyst for the single pot multicomponent reaction (Table 3, entry 1), we have increased the amount of all the reactants by a factor of 10 and achieved about 92% of the cycloaddition product, 1-benzyl-4-phenyl-1*H*-1,2,3-triazole **(3a)**, in 9h. After the first run, we have recovered the copper-polymer composite and used for the recyclability test to find out the performance of the reused catalyst. At the end of first cycle a yield of 92% of the coupled product **(3a)** has been achieved and that took about 8 h, which is one hour less than the original single pot multicomponent reaction. The reason for the improved performance can be addressed in terms of nanoparticle formation (as discussed before). At the end of the fifth cycle, a yield of 76% of cycloaddition product was achieved at 8 h (Figure 7).

The *in situ* generated Cu(I) plays the catalytic role for the title reaction. Polyaniline acts as a ligand to coordinate to the Cu(I) species which involves the formation of a Cu(I)-acetylidine complex through the coordination with alkyne followed by the addition with the azide group to give 1,2,3-triazole. It is also important to mention that in the present study we found that all reactions were highly regioselective towards the formation of 1,4-disubstituted triazoles with a wide range of diversely substituted terminal alkynes and azides under the optimized conditions.

## Conclusion

In this report, we have presented an interesting method where the catalyst formation occurs in the reaction medium that prevents the catalyst from the environmental degradation. The elimination of the separate catalyst synthesis step may be economical by saving the time as well as the solvents. Aniline was used as one of the reactant components so there was no requirement of adding additional base for this reaction as recommended by the original protocol of the azide-alkyne cycloaddition (Click) reaction. Furthermore, the catalyst can be readily recovered by filtration and efficiently used for the similar reaction without any significant loss of catalytic activity. The operational simplicity and the purity (regeioslectivity) of the products make this method attractive for wide range of applications.

## Methods

### General procedure for azide and alkyne cycloaddition reaction

In a 25 mL round bottom flask, alkyne (1 equiv.) and azide (1 equiv., benzyl azide/*o*-bromo benzyl azide) were taken and dissolved in 5 ml methanol. To this reaction mixture 1 ml of 0.1 M of aniline in methanol was added and stirred at room temperature. To this solution 5 ml of 0.01 M solution of CuSO_4_, 5H_2_O (in water) was added drop wise. A green colourization was appeared during the addition of the CuSO_4_, 5H_2_O. The reaction mixture was stirred for 7 h at room temperature and progress of the reaction was monitored using thin layer chromatography technique. After completion, the reaction mixture was filtered and the residue was dissolved with methanol. The remaining solid catalyst was recovered, dried and reused for the recyclability experiment. The methanol was evaporated from the filtrate and extracted with ethyl acetate, washed with water and dried over anhydrous sodium sulphate. Combined organic layer was concentrated in vacuum to give the corresponding triazoles which was pure enough or was purified by column chromatography technique. The products were characterised by spectroscopic analysis or by comparison of the spectroscopic data with those described in the literature.

### General procedure for multicomponent azide-alkyne cycloaddition

The above mentioned procedure was followed in a 25 mL round bottom flask using alkyl halide (1 equiv.), NaN_3_ (1 equiv.) and an alkyne (1 equiv.) in methanol (5.0 mL) in the presence of 1 ml of 0.1 M of aniline. To this solution 5 ml of 0.01 M solution of CuSO_4_, 5H_2_O (in water) was added drop wise for the cycloaddition reaction.

## Supplementary Material

Supplementary Information Supporting Information

## Figures and Tables

**Table 1:**
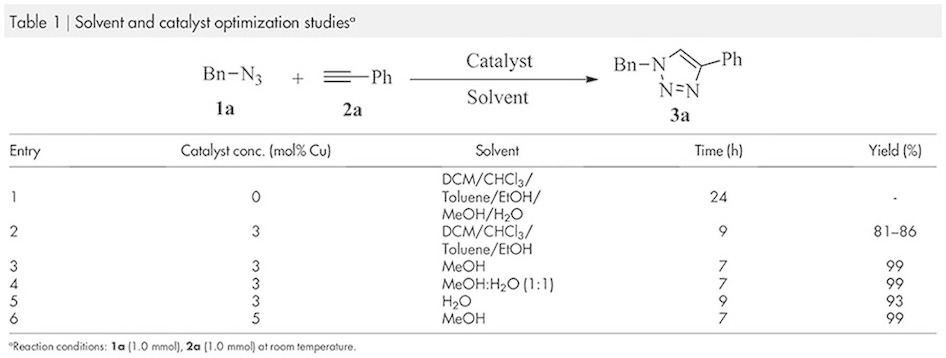
Solvent and catalyst optimization studies^a^.

**Table 2:**
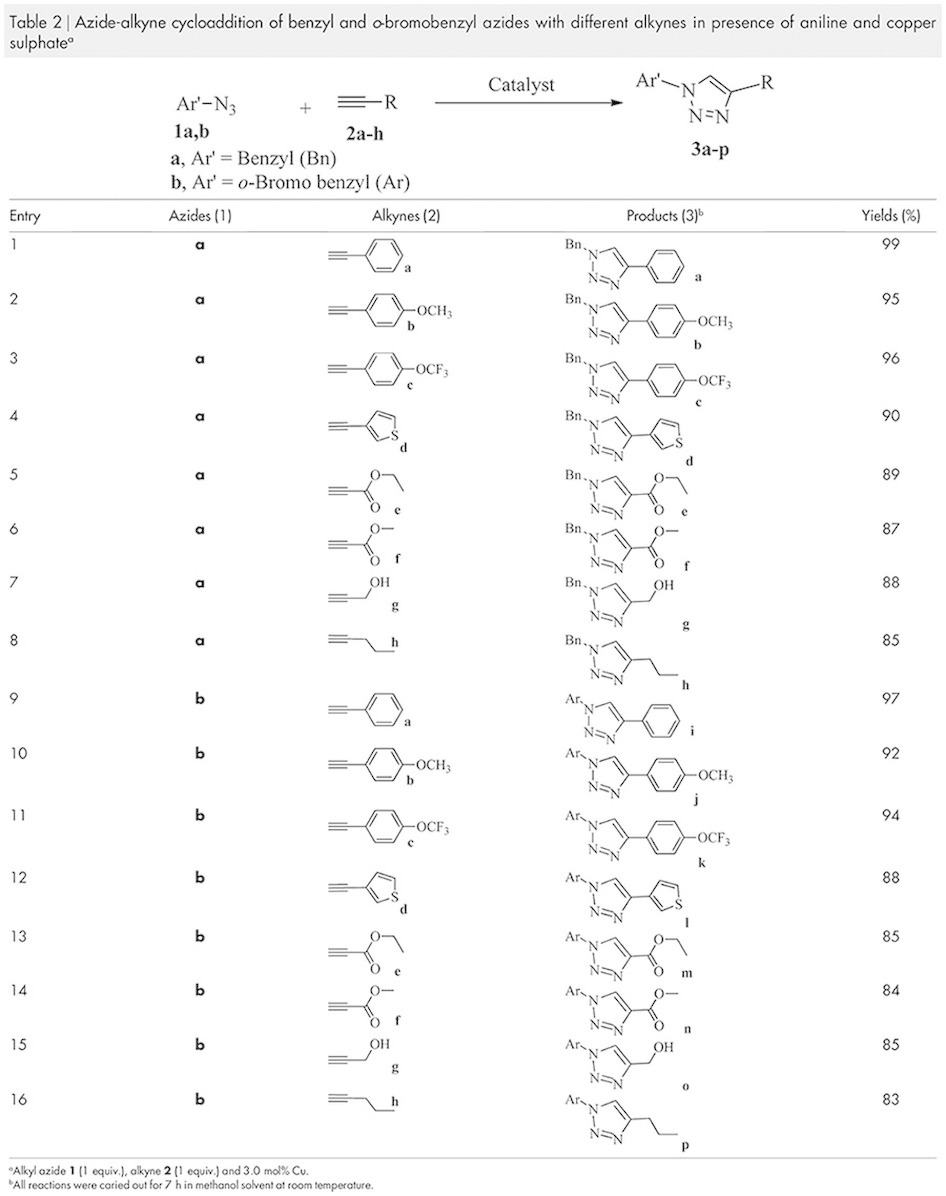
Azide-alkyne cycloaddition of benzyl and o-bromobenzyl azides with different alkynes in presence of aniline and copper sulphate^a^.

**Table 3:**
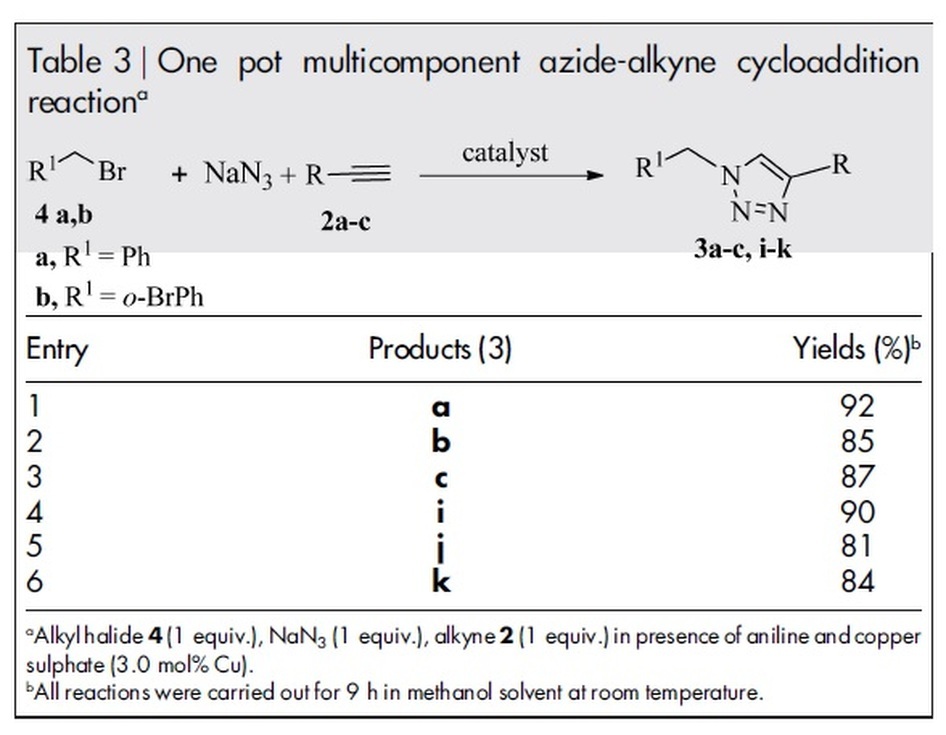
One pot multicomponent azide-alkyne cycloaddition reaction^a^.

## References

[b1] Katritzky A. R. & Pozharskii A. F. [Reactivity of Heterocycles.] Handbook of Heterocyclic Chemistry [Katritzky A. R., & Pozharskii A. F., eds. , (eds.)] [272–274] (Elsevier Science Ltd, Oxford, U.K., 2000).

[b2] Eicher T. & Hauptmann S. The Chemistry of Heterocycles [Eicher T., & Hauptmann S., eds. (eds.)] [1–554] (Wiley-VCH, 2003).

[b3] Tron G. C. *et al.* Click chemistry reactions in medicinal chemistry: applications of the 1,3-dipolar cycloaddition between azides and alkynes. Med. Res. Rev. 28, 278–308 (2008). 17763363 10.1002/med.20107

[b4] Lee T. *et al.* Synthesis and Evaluation of 1,2,3-Triazole Containing Analogues of the Immunostimulant α-GalCer. J. Med. Chem. 50, 585–589 (2007). 17266209 10.1021/jm061243q

[b5] Bastero A., Font D. & Pericàs M. A. Assessing the Suitability of 1,2,3-Triazole Linkers for Covalent Immobilization of Chiral Ligands: Application to Enantioselective Phenylation of Aldehydes. J. Org. Chem. 72, 2460–2468 (2007). 17346086 10.1021/jo0624952

[b6] Wamhoff H. Comprehensive Heterocyclic Chemistry [Katritzky A. R., & Rees C. W., eds. (eds.)] [669–732] (Pergamon, Oxford, 1984).

[b7] Huisgen R. 1, 3-Dipolar Cycloaddition Chemistry [Padwa A., ed. (ed.)] [1–176] (Wiley, New York, 1984).

[b8] Rostovtsev V. V., Green L. G., Fokin V. V. & Sharpless K. B. A Stepwise Huisgen Cycloaddition Process: Copper(I)-Catalyzed Regioselective “Ligation” of Azides and Terminal Alkynes. Angew. Chem., Int. Ed. 41, 2596–2599 (2002). 10.1002/1521-3773(20020715)41:14<2596::AID-ANIE2596>3.0.CO;2-4 12203546

[b9] Tornøe C. W., Christensen C. & Meldal M. Peptidotriazoles on Solid Phase: [1,2,3]-Triazoles by Regiospecific Copper(I)-Catalyzed 1,3-Dipolar Cycloadditions of Terminal Alkynes to Azides. J. Org. Chem. 67, 3057–3064 (2002). 11975567 10.1021/jo011148j

[b10] Rodionov V. O., Presolski S. I., Gardinier S., Lim Y.-H. & Finn M. G. Benzimidazole & Related Ligands for Cu-Catalyzed Azide-Alkyne Cycloaddition. J. Am. Chem. Soc. 129, 12696–12704 (2007). 17914816 10.1021/ja072678l

[b11] Girard C. *et al.* Reusable Polymer-Supported Catalyst for the [3+2] Huisgen Cycloaddition in Automation Protocols. Org. Lett. 8, 1689–1692 (2006). 16597142 10.1021/ol060283l

[b12] Chassaing S., Kumarraja M., Sido A. S. S., Pale P. & Sommer J. Click Chemistry in CuI-zeolites: The Huisgen [3 + 2]-Cycloaddition. Org. Lett. 9, 883–886 (2007). 17286410 10.1021/ol0631152

[b13] Molteni G., Bianchi C. L., Marinoni G., Santo N. & Ponti A. Cu/Cu-oxide nanoparticles as catalyst in the “click” azide–alkyne cycloaddition. New J. Chem. 30, 1137–1139 (2006).

[b14] Himo F. *et al.* Copper(I)-Catalyzed Synthesis of Azoles. DFT Study Predicts Unprecedented Reactivity and Intermediates. J. Am. Chem. Soc. 127, 210–216 (2005). 15631470 10.1021/ja0471525

[b15] Kim J. Y., Park J. C., Kang H., Song H. & Park K. H. CuO hollow nanostructures catalyze [3 + 2] cycloaddition of azides with terminal alkynes. Chem. Commun. 46, 439–441 (2010). 10.1039/b917781g 20066318

[b16] Wang D. *et al.* Solvent-free synthesis of 1,4-disubstituted 1,2,3-triazoles using a low amount of Cu(PPh_3_)_2_NO_3_ complex. Green Chem. 12, 2120–2123 (2010).

[b17] Zhang Z. *et al.* Stabilized Copper(I) Oxide Nanoparticles Catalyze Azide-Alkyne Click Reactions in Water. Adv. Synth. Catal. 352, 1600–1604 (2010).

[b18] Shao C. *et al.* Copper(I) oxide and benzoic acid ‘on water’: a highly practical and efficient catalytic system for copper(I)-catalyzed azide–alkyne cycloaddition. Tetrahedron Lett. 52, 3782–3785 (2011).

[b19] Rad M. N. S. *et al.* Doped Nano-Sized Copper(I) Oxide (Cu_2_O) on Melamine-Formaldehyde Resin: a Highly Efficient Heterogeneous Nano Catalyst for ‘Click’ Synthesis of Some Novel 1H-1,2,3-Triazole Derivatives Having Antibacterial Activity. Helv. Chim. Acta. 96, 688–701 (2013).

[b20] Wang K. *et al.* Cu_2_O acting as a robust catalyst in CuAAC reactions: water is the required medium. Green Chem. 13, 562–565 (2011).

[b21] Jiang Y. *et al.* Cu(OAc)_2_·H_2_O/NH_2_NH_2_·H_2_O: an efficient catalyst system that in situ generates Cu_2_O nanoparticles and HOAc for Huisgen click reactions. RSC Adv. 4, 1010–1014 (2014).

[b22] Rodionov V. O., Presolski S. I., Díaz D. D., Fokin V. V. & Finn M. G. Ligand-Accelerated Cu-Catalyzed Azide−Alkyne Cycloaddition: A Mechanistic Report. J. Am. Chem. Soc. 129, 12705–12712 (2007). 17914817 10.1021/ja072679d

[b23] Granot E., Katz E., Basnar B. & Willner I. Enhanced Bioelectrocatalysis Using Au Nanoparticle/Polyaniline Hybrid Systems in Thin Films and Microstructured Rods Assembled on Electrodes. Chem. Mater. 17, 4600–4609 (2005).

[b24] Feng X., Mao C., Yang G., Hou W. & Zhu J. J. Polyaniline/Au Composite Hollow Spheres: Synthesis, Characterization, and Application to the Detection of Dopamine. Langmuir 22, 4384–4389 (2006). 16618191 10.1021/la053403r

[b25] Tseng R. J., Huang J., Ouyang J., Kaner R. B. & Yang Y. Polyaniline Nanofiber/Gold Nanoparticle Nonvolatile Memory. Nano Lett. 5, 1077–1080 (2005). 15943446 10.1021/nl050587l

[b26] Dong H. *et al.* One-Pot Synthesis of Robust Core/Shell Gold Nanoparticles. J. Am. Chem. Soc. 130, 12852–12853 (2008). 18763773 10.1021/ja8038097

[b27] Scalzullo S. *et al.* Polymer-encapsulated metal nanoparticles: optical, structural, micro-analytical and hydrogenation studies of a composite material. Nanotechnology 19, 075708 (2008). 21817656 10.1088/0957-4484/19/7/075708

[b28] Islam R. *et al.* Metal–Polymer Hybrid Material as a Catalyst for the Heck Coupling Reaction Under Phosphine-Free Conditions. Synth. Commun. 41, 3561–3572 (2011).

[b29] Islam R. *et al.* Conjugated polymer stabilized palladium nanoparticles as a versatile catalyst for Suzuki cross-coupling reactions for both aryl and heteroaryl bromide systems. Catal. Sci. Tech. 1, 308–315 (2011).

[b30] Islam R. *et al.* In-situ synthesis of a palladium-polyaniline hybrid catalyst for a Suzuki coupling reaction. J. Organomet. Chem. 696, 2206–2210 (2011).

[b31] Islam R., Witcomb M., Scurrell M., Otterlo W. & Mallick K. In situ synthesis of a Pd--poly (1, 8-diaminonaphthalene) nanocomposite: An efficient catalyst for Heck reactions under phosphine-free conditions. Catal. Commun. 12, 116–121 (2010).

[b32] Islam R., Mahato S., Shukla S., Witcomb M. & Mallick K. Palladium–Poly(3-aminoquinoline) Hollow-Sphere Composite: Application in Sonogashira Coupling Reactions. ChemCatChem, 5, 2453–2461 (2013).

[b33] Mallick K., Witcomb M. & Scurrell M. Fabrication of a nanostructured gold-polymer composite material. Euro. Phys. J. E. 20, 347–353 (2006). 10.1140/epje/i2006-10023-3 16871368

[b34] Mallick K., Witcomb M. & Scurrell M. Formation of palladium nanoparticles in poly (o-methoxyaniline) macromolecule fibers: An in-situ chemical synthesis method. Euro. Phys. J. E. 19, 149–154 (2006). 10.1140/epje/e2006-00027-2 16525766

[b35] Mallick K., Witcomb M., Dinsmore A. & Scurrell M. Fabrication of a metal nanoparticles and polymer nanofibers composite material by an in situ chemical synthetic route. Langmuir 21, 7964–7967 (2005). 16089406 10.1021/la050534j

[b36] Mallick K., Witcomb M., Dinsmore A. & Scurrell M. Polymerization of aniline by auric acid: formation of gold decorated polyaniline nanoballs. Macromol. Rapid Commun. 26, 232–235 (2005).

[b37] Mallick K., Witcomb M., Erasmus R. & Strydom A. Low-temperature magnetic property of polymer encapsulated gold nanoparticles. J. Appl. Phys. 106, 074303 (2009).

[b38] Mallick K., Witcomb M., Scurrell M. & Strydom A. Optical, microscopic and low temperature electrical property of one-dimensional gold–polyaniline composite networks. J. Phys. D, Appl. Phys. 42, 095409 (2009).

[b39] Stejskal J., Trchová M., Prokeš J. & Sapurina I. Brominated Polyaniline. Chem. Mater. 13, 4083–4086 (2001).

[b40] Pillalamarri S. K., Blum F. D., Tokuhiro A. T. & Bertino M. F. One-Pot Synthesis of Polyaniline−Metal Nanocomposites. Chem. Mater. 17, 5941–5944 (2005).

[b41] Alonso F., Moglie Y., Radivoy G. & Yus M. Copper nanoparticles in click chemistry: an alternative catalytic system for the cycloaddition of terminal alkynes and azides. Tetrahedron Lett. 50, 2358–2362 (2009).

[b42] Baqi Y. & Müller C. E. Convergent Synthesis of the Potent P2Y Receptor Antagonist MG 50-3-1 Based on a Regioselective Ullmann Coupling Reaction. Molecules 17, 2599–2615 (2012). 22391596 10.3390/molecules17032599 PMC6268193

[b43] Alonso F., Moglie Y., Radivoy G. & Yus M. Copper-Catalysed multicomponent Click Synthesis of 5-Alkynyl 1,2,3-Triazoles under Ambient Conditions. Synlett 15, 2179–2182 (2012).

[b44] Sarkar A., Mukherjee T. & Kapoor S. PVP-Stabilized Copper Nanoparticles: A Reusable Catalyst for “Click” Reaction between Terminal Alkynes and Azides in Nonaqueous Solvents. J. Phys. Chem. C 112, 3334–3340 (2008).

[b45] Bock V. D., Hiemstra H. & Maarseveen J. H. CuI-Catalyzed Alkyne–Azide “Click” Cycloadditions from a Mechanistic and Synthetic Perspective. Eur. J. Org. Chem. 1, 51–68 (2006).

[b46] Lipshutz B. H., Frieman B. A. & Tomaso Jr A. E. Copper-in-Charcoal (Cu/C): Heterogeneous, Copper-Catalyzed Asymmetric Hydrosilylations. Angew. Chem., Int. Ed. 45, 1259–1264 (2006). 10.1002/anie.200503149 16425315

[b47] Reddy K. R., Rajgopal K. M. & Kantam M. L. Copper(II)-Promoted Regioselective Synthesis of 1,4-Disubstituted 1,2,3-Triazoles in Water. Synlett 6, 957–959 (2006).

[b48] Garret C. E. & Prasad K. The Art of Meeting Palladium Specifications in Active Pharmaceutical Ingredients Produced by Pd-Catalyzed Reactions. Adv. Synth. Cat. 346, 889–900 (2004).

[b49] Phan N. T. S., Van Der Sluys M. & Jones C. W. On the Nature of the Catalytic Species in Palladium Catalyzed Heck and Suzuki Couplings: Homogeneous or Heterogeneous Catalysis, a Critical Review. Adv. Synth. Cat. 348, 609–679 (2006).

