# Supplementary material for: Polymer immobilized Cu(I) formation and azide-alkyne cycloaddition: A one pot reaction
Source: Sci Rep. 2015 May 12;5:9632. doi: 10.1038/srep09632 (PMC4603298; doi:10.1038/srep09632)
Supplement: Supplementary Information — Supporting Information [file srep09632-s1.doc]

**Supporting Information**

**Polymer immobilized Cu(I) formation and azide-alkyne cycloaddition: A one pot reaction**

**Rafique Ul Islam, Abu Taher, Meenakshi Choudhary, Samarjeet Siwal and Kaushik Mallick***

Department of Chemistry, University of Johannesburg, Post Box 524, Auckland Park 2006,

Johannesburg, South Africa.

**Table 2: Entry 1**

**Compound 3a[**^1,2]^: 1-Benzyl-4-phenyl-1*H*-1,2,3-triazole

White solid, yield: 233 mg, 99%; mp: 136-138^o^C (lit.**[**^2]^ mp: 128-130ºC)

**^1^H NMR (400 MHz, CDCl_3_, δ, ppm):** 5.55(s, 2H, -NCH_2_), 7.23-7.39(m, 8H, ArH), 7.64(s, 1H, -C=CH), 7.77-7.78(d, *J* = 7.6 Hz, 2H, ArH); **^13^C NMR (100 MHz, CDCl_3_, δ, ppm):** 54.20, 119.48, 125.66, 128.03, 128.15, 128.77, 129.12, 130.44, 134.62, 148.16.

**Table 2: Entry 2**

**Compound 3b[**^2,3]^: 1-Benzyl-4-(4-methoxyphenyl)-1*H*-1,2,3-triazole

White solid, yield: 252 mg, 95%; mp: 154-156^o^C (lit.**[**^2]^ mp: 143-144^o^C)

**^1^H NMR (400 MHz, CDCl_3_, δ, ppm**): 3.79(s, 3H, -OCH_3_), 5.55(s, 2H, -NCH_2_), 6.88-6.90(m, 2H, ArH), 7.23-7.34(m, 5H, ArH), 7.68-7.73(m, 3H, ArH); **^13^C NMR (100 MHz, CDCl_3_, δ, ppm):** 54.87, 55.33, 114.34, 122.00, 127.30, 128.25, 128.88, 129.17, 134.32, 159.92.

**Table 2: Entry 3**

**Compound 3c:** 1-Benzyl-4-(4-(trifluoromethoxy)phenyl)-1*H*-1,2,3-triazole

White solid, yield: 306 mg, 96%, mp: 132-133^o^C

**^1^H NMR (400 MHz, CDCl_3_, δ, ppm):** 5.61(s, 2H, -NCH_2_), 7.26-7.41(m, 7H, ArH), 7.76(s, 1H), 7.85-7.87(m, 2H, ArH); **^13^C NMR (100 MHz, CDCl_3_, δ, ppm):** 30.87, 54.33, 121.31, 127.19, 128.14, 128.93, 129.06, 129.21, 134.37, 149.08.

**Table 2: Entry 4:**

**Compound 3d:** 1-Benzyl-4-(thiophen-3-yl)-1*H*-1,2,3-triazole

White solid, yield: 217 mg, 90%; mp: 149-151^o^C

**^1^H NMR (400 MHz, CDCl_3_, δ, ppm):** 5.52(s, 2H, -NCH_2_), 7.25-7.27(m, 2H, ArH), 7.31-7.37(m, 4H, ArH), 7.38-7.39(dd, *J* = 1.2, 5.2 Hz, 1H, ArH), 7.55(s, 1H, -C=CH), 7.62-7.63(dd, *J* = 1.2, 7.2 Hz, 1H, ArH); **^13^C NMR (100 MHz, CDCl_3_, δ, ppm):** 54.22, 119.42, 121.2, 125.82, 126.35, 128.07, 128.82, 129.19, 131.74, 134.68, 144.57.

**Table 2: Entry 5**

**Compound 3e[**^1c,2]^: Ethyl 1-benzyl-1*H*-1,2,3-triazole-4-carboxylate

Pale yellow solid, yield: 205 mg, 89%; mp: 92-94^o^C (lit.**[**^2]^ mp: 83-85ºC.)

**^1^H NMR (400 MHz, CDCl_3_, δ, ppm):** 1.31-1.35(t, *J* = 7.6 Hz, 3H, CH_3_), 4.32-4.37(q, *J* = 8.2 Hz, 2H, -CH_2_), 5.53(s, 2H, -NCH_2_), 7.24-7.34(m, 5H, ArH), 7.94(s, 1H, -C=CH); **^13^C NMR (100 MHz, CDCl_3_, δ, ppm):** 14.20. 54.39, 61.20, 127.25, 128.17, 129.03, 129.22, 133.69, 140.55, 160.59.

**Table 2: Entry 6**

**Compound 3f:** Methyl 1-benzyl-1*H*-1,2,3-triazole-4-carboxylate

White solid, yield: 189 mg, 87%; mp: 113-115^o^C

**^1^H NMR (400 MHz, CDCl_3_, δ, ppm):** 3.89(s, 3H, -OCH_3_), 5.55(s, 2H, -NCH_2_), 7.24-7.27(m, 2H, ArH), 7.35-7.37(m, 3H, ArH), 7.96(s, 1H, -C=CH); **^13^C NMR (100 MHz, CDCl_3_, δ, ppm):** 52.16, 54.50, 128.27, 129.16, 129.31, 133.61, 161.08.

**Table 2: Entry 7**

**Compound 3g[**^1a]^: (1-Benzyl-1*H*-1,2,3-triazol-4-yl)methanol

White solid, yield: 166 mg, 88%; mp: 81-83^o^C (lit.**[**^1a]^ mp: 76–78ºC­)

**^1^H NMR (400 MHz, CDCl_3_, δ, ppm):** 4.17(s, 1H, -OH), 4.76(s, 2H, -OCH_2_), 5.52(s, 2H, -NCH_2_), 7.28-7.29(m, 2H, ArH), 7.36(bs, 3H, ArH), 7.54(s, 1H, -C=CH); **^13^C NMR (100 MHz, CDCl_3_, δ, ppm**): 54.12, 55.71, 122.21, 127.99, 128.94, 129.00, 134.32, 148.22.

**Table 2, Entry 8**

**Compound 3h[**^4]^**:** 1-Benzyl-4-propyl-1*H*-1,2,3-triazole

Liquid compound, yield: 171 mg, 85%.

**^1^H NMR (400 MHz, CDCl_3_, δ, ppm):** 0.93-0.97(t, J = 7.6 Hz, 3H, -CH_3_), 1.63-1.72(m, 2H, -CH_2_), 2.66-2.69(t, *J* = 7.6 Hz, 2H, -CH_2_), 5.50(s, 2H, -NCH_2_), 7.24-7.27(m, 3H, ArH), 7.35-7.37(m, 3H, ArH); **^13^C NMR (100 MHz, CDCl_3_, δ, ppm):** 13.62, 22.50, 27.53, 53.86, 120.56, 127.81, 128.46, 128.90, 134.87, 148.48.

**Table 2: Entry 9**

**Compound 3i[**^1c,5]^**:** 1-(2-Bromobenzyl)-4-phenyl-1*H*-1,2,3-triazole

White solid, yield: 304 mg, 97%; mp: 110-112^o^C (lit.**[**^1c]^ mp: 103-104^o^C)

**^1^H NMR (400 MHz, CDCl_3_, δ, ppm):** 5.33(s, 2H, N-CH_2_), 7.19-7.21(d, *J* = 7.6 Hz, 1H, ArH), 7.25-7.27(d, *J* = 7.6 Hz, 1H, ArH), 7.31-7.35(t, *J* = 7.6 Hz, 1H, ArH), 7.46-7.54(m, 3H, ArH), 7.56-7.59(m, 1H, ArH), 7.70-7.72(d, *J* = 8.0 Hz, 2H, ArH); **^13^C NMR (100 MHz, CDCl_3_, δ, ppm):** 52.08, 119.89, 123.30, 125.86, 127.74, 128.79, 128.84, 129.11, 130.07, 130.18, 132.33, 132.94, 148.18.

**Table 2: Entry 10**

**Compound 3j:** 1-(2-Bromobenzyl)-4-(4-methoxyphenyl)-1*H*-1,2,3-triazole

White solid, yield: 315 mg, 92%; mp: 156-158^o^C

**^1^H NMR (400 MHz, CDCl_3_, δ, ppm):** 3.80(s, 3H, -OCH_3_), 5.66(s, 2H, -NCH_2_), 6.90-6.92(d, *J* = 8.4 Hz, 2H, ArH), 7.13-7.29(m, 3H, ArH), 7.58-7.60(d, *J* = 7.6 Hz, 1H, ArH), 7.21-7.73(d, *J* = 8.4 Hz, 3H, ArH); **^13^C NMR (100 MHz, CDCl_3_, δ, ppm):** 53.99, 55.27, 114.25, 119.30, 122.62, 123.40, 127.14, 128.22, 130.31, 130.39, 133.16, 134.07, 159.78.

**Table 2: Entry 11**

**Compound 3k:** 1-(2-Bromobenzyl)-4-(4-(trifluoromethoxy)phenyl)-1*H*-1,2,3-triazole

White solid, yield: 374 mg, 94%; mp: 90-92^o^C

**^1^H NMR (400 MHz, CDCl_3_, δ, ppm):** 5.66(s, 2H, -NCH_2_), 7.21-7.80(m, 9H, ArH); **^13^C NMR (100 MHz, CDCl_3_, δ, ppm):** 53.97, 54.21, 120.00, 121.29, 121.70, 123.48, 127.11, 128.26, 129.21, 130.37, 130.49, 133.26, 134.01, 149.00.

**Table 2: Entry 12**

**Compound 3l:** 1-(2-Bromobenzyl)-4-(thiophen-3-yl)-1*H*-1,2,3-triazole

White solid, yield: 281 mg, 88%; mp: 104-106^o^C

**^1^H NMR (400 MHz, CDCl_3_, δ, ppm):** 5.62(s, 2H, -NCH_2_), 7.09-7.11(dd, *J* = 1.6, 7.6 Hz, 1H, ArH), 7.15-7.20(dt, *J* = 2.0, 7.6 Hz, 1H, ArH), 7.23-7.27(m, 1H, ArH), 7.30-7.32(m, 1H, ArH), 7.39-7.40(dd, *J* = 1.2, 5.2 Hz, 1H, ArH), 7.56-7.58(dd, *J* = 1.2, 7.6 Hz, 1H, ArH), 7.62-7.63(dd, *J* = 1.2, 2.8 Hz, 1H, ArH), 7.66(s, 1H, -C=CH); **^13^C NMR (100 MHz, CDCl_3_, δ, ppm):** 53.67, 119.63, 121.08, 123.24, 125.69, 126.21, 128.11, 130.05, 130.26, 131.56, 133.06, 134.09, 144.12.

**Table 2: Entry 13**

**Compound 3m:** Ethyl 1-(2-bromobenzyl)-1*H*-1,2,3-triazole-4-carboxylate

White solid, yield: 263 mg, 85%; mp: 87-89^o^C

**^1^H NMR (400 MHz, CDCl_3_, δ, ppm):** 1.34-1.38(t, *J* = 7.2, 14.0 Hz, 3H, -CH_3_), 4.35-4.40(q, *J* = 7.2, 14.4 Hz, 2H, -CH_2_-), 5.68(s, 2H, -NCH_2_-), 7.20-7.25( m, 2H, ArH), 7.29-7.33(t, *J* = 7.2, 14.4 Hz, 1H, ArH), 7.60-7.62(d, *J* = 7.6 Hz, 1H, ArH), 8.04(s, 1H, -C=CH); **^13^C NMR (100 MHz, CDCl_3_, δ, ppm):** 14.28, 54.14, 61.33, 123.76, 127.55, 128.37, 130.74, 130.85, 133.25, 133.43, 140.52, 160.67.

**Table 2: Entry 14**

**Compound 3n:** Methyl 1-(2-bromobenzyl)-1*H*-1,2,3-triazole-4-carboxylate

White solid, yield: 248 mg, 84%; mp: 120-122^o^C

**^1^H NMR (400 MHz, δ, ppm):** 3.89(s, 3H, -CH_3_), 5.67(s, 2H, -NCH_2_), 7.20-7.23(m, 2H, ArH), 7.28-7.30(m, 1H), 7.58-7.60(m, 1H, ArH), 8.08 (s, 1H, -C=CH); **^13^C NMR (100 MHz, δ, ppm):** 52.22, 54.27, 123.80, 128.04, 128.37, 130.55, 130.82, 130.90, 133.14, 133.45, 161.16.

**Table 2: Entry 15**

**Compound 3o:** (1-(2-Bromobenzyl)-1*H*-1,2,3-triazol-4-yl) methanol

White solid, yield: 227 mg, 85%; mp: 124-126^o^C

**^1^H NMR (400 MHz, CDCl_3_, δ, ppm):** 3.12(bs, 1H, -OH), 4.74(s, 2H, -OCH_2_), 5.61(s, 2H, -NCH_2_), 7.13-7.29(m, 3H, ArH), 7.56-7.59(m, 2H, ArH); **^13^C NMR (100 MHz, CDCl_3_, δ, ppm):** 53.90, 56.27, 122.13, 123.51, 124.0, 128.22, 130.46, 133.22, 133.93, 148.05.

**Table 2: Entry 16**

**Compound 3p:** 1-(2-Bromobenzyl)-4-propyl-1*H*-1,2,3-triazole

Yield: 232 mg, 83%; liquid compound

**^1^H NMR (400 MHz, δ, ppm):** 0.87-0.91(t, *J* = 3.6 Hz, 3H, -C*H*_3_), 1.58-1.67(m, 2H, -CH_2_-C*H*_2_-CH_3_), 2.61-2.64(t, *J* = 3.6 Hz, 2H, -C*H*_2_-CH_2_-CH_3_), 5.56(s, 2H, -NCH_2_), 7.01-7.041(dd, *J* = 1.6, 7.6 Hz, 1H, ArH), 7.12-7.16(dt, *J* = 2, 7.6, 15.6 Hz, 1H, ArH), 7.20-7.25(dt, *J* = 1.2, 7.6, 15.2 Hz, 1H, ArH), 7.27(s, 1H, C=C*H*), 7.53-7.55(dd, *J* = 1.6, 8 Hz, 1H, ArH); **^13^C NMR (100 MHz, δ, ppm):** 13.62, 22.5, 27.54, 53.49, 120.93, 123.14, 128.02, 129.94, 130.6, 132.98, 134.43, 148.42.

**References**:

[1] F. Alonso, Y. Moglie, G. Radivoy, M. Yus, *Eur. J. Org. Chem.*, **2010**, 1875–1884; \

[2] V. V. Rostovtsev, L. G. Green, V. V. Fokin, K. B. Sharpless, *Angew. Chem., Int. Ed.,* **2002**, *41*, 2596-2599;

[3] C. Shao, R. Zhu, S. Luo, Q. Zhang, X. Wang, Y. Hu, *Tetrahedron Lett.,* **2011**, *52,* 3782-3785.

[4] L. Campbell-Verduyna, L. Mirfeizib, R. A. Dierckxb, P. H. Elsingab, Ben L. Feringaa,

*Chem. Commun.* **2009**, 2139-2141.

[5] V. O. Rodionov, S. I. Presolski, S. Gardinier, Y.-H. Lim, M. G. Finn, *J. Am. Chem. Soc*.,

**2007**, *129*, 12696-12704.

[6] J. –A. Shin, Y. –G. Lim, K. –H. Lee*, J. Org, Chem*, **2012**, *77*, 4117-4122.

[7] S. T. Abu-Orabi, M. A. Atfah, I. Jibril, F. M. Maríi, A. A. –S. Ali, *J. Heterocyclic Chem.* **1989**, *26*, 1461-1468.
